# The history of GM crops in Italy: After two decades of a de facto ban, the engagement of farmers and scientists has prompted the Italian government to allow field-testing of NGT/TEA plants again

**DOI:** 10.1038/s44319-024-00330-3

**Published:** 2024-11-27

**Authors:** Roberto Defez, Maria Chiara Errigo, Giulia Formici, Lucia Scaffardi, Eleonora Sirsi, Fabio Fornara, Vittoria Brambilla

**Affiliations:** 1 Institute of Biosciences and Bioresources (IBBR), CNR, Napoli, Italy; 2 Department of Law, Politics and International Studies, University of Parma, Parma, Italy; 3 Department of Law, University of Pisa, Pisa, Italy; 4 Department of Biosciences, University of Milan, Milan, Italy; 5 Department of Agricultural and Environmental Sciences - Production, Landscape, Agroenergy, University of Milan, Milan, Italy

**Keywords:** Biotechnology & Synthetic Biology, Economics, Law & Politics, Plant Biology

## Abstract

The fate of GM crops in Italy—from endorsement to rejection back to wary acceptance—may hold lessons for other EU member states’ policies on biotechnology.

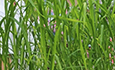

During the past decades, Italy has adopted divergent approaches to using biotechnologies in plant breeding and agriculture. The governments’ policies have ranged from initial endorsement and support to a state of relative paralysis and is currently swinging back to support New Genetic Technologie (NGTs). Pressure from Italian scientists and several lawsuits filed in the Court of Justice of the European Union by an Italian farmer have prompted the government’s re-evaluation of EU policies on genetically modified organisms (GMOs). These events and legal precedents may not provide an indication of future trends regarding the use of biotechnology in agriculture in the EU, but could inspire a renewed sense of collaboration among scientists, legislators, and agricultural entrepreneurs.

## The history of using new technologies for plant breeding in Italy

The first GM crops in Europe were field-tested mainly in Italy and France between 1992 and 2004. The modified traits included over a dozen tolerance or resistance characteristics within 24 different plant species. Italy has in fact a long tradition in plant breeding using the latest technologies. In 1930s, geneticist Nazareno Strampelli developed wheat varieties with reduced size by crossing bread wheat with exotic semi-dwarf cultivars even before Norman Borlaug’s famous work. Gian Tommaso Scarascia Mugnozza introduced the application of plant mutagenesis by ionizing radiation and Francesco Salamini led breeding into the era of molecular genetics and biotechnology.

Italian politics initially showed interest in biotechnology with public investments into research and the ICGEB in Trieste, and the development of an agro-biotechnology private sector (Sirsi, 2017). However, at the turn of the millennium, new political developments paralyzed the use of recombinant DNA technologies and halted plant research. The reasons for this sudden stop included a new environmental movement, public concerns about new technologies and contamination of natural ecosystems, and consumer aversion to multinational food companies. Economic interests accompanied these public concerns: the technological lag of European breeding companies, the potential impact on the production of agrochemicals in Europe due to the new genetic traits and the competition for agricultural subsidies from the EU.

“Italian politics initially showed interest in biotechnology with public investments into research and the ICGEB in Trieste, and the development of an agro-biotechnology private sector.”

These political developments and public opinion created an atmosphere of apprehension and skepticism about technological innovation in agriculture. In this climate, the Italian Minister of Health signed a temporary ban on the cultivation of Bt corn in 1997, based on concerns that the insertion of the Bt-endotoxin gene could generate resistance in non-target insects, potentially causing ecosystem disruption. Three years later, the Italian government temporarily suspended the marketing of Bt corn flours in Italy, based on Article 12(1) of EU Regulation 1997/258/EC, under which a EU member state can take national safeguard measures, as a result of new information or a reassessment of existing information, when the government has reason to believe that the use of a food or food ingredient endangers human health or the environment. This decree, known as the Amato Decree, was designed to regulate the import of GM food, but the ban affected only products that contained traces of GM plants’ DNA. For this reason, Bt corn was banned but not herbicide-resistant rapeseed because the latter was consumed in the form of oil that is nearly free of DNA. The Amato Decree was challenged before the Italian Administrative Court, initiating a protracted legal dispute, which involved both the Italian courts and the Court of Justice of the European Union (CJEU).

These political decisions triggered the reaction of Italian scientists from various disciplines who, on February 13, 2001, gathered in the library of the Italian Chamber of Deputies to oppose what they perceived as obscurantist policies on GMOs that badly affected plant research (Meldolesi, 2001). This event was widely covered by the Italian the media and had a significant impact on the political debate. Meanwhile, at the European Union (EU), the need to regulate the use of GMOs and the increased public skepticism about it led to the adoption of Directive 2001/18/EC, characterized by a strong precautionary approach. In Italy, Directive 2001/18/EC was implemented by Legislative Decree No. 224/2003 and later modified with Legislative Decree No. 227/2016. Unlike the EU Directive, the Italian Decree introduced a mandatory risk assessment for the release of GM plants for scientific purposes, thereby ignoring the criticism by a large number of scientists. Above all, it introduced sanctions of up to three years imprisonment for anyone planting a GM crop without prior authorization.

“Unlike the EU Directive, the Italian Decree introduced a mandatory risk assessment for the release of GM plants for scientific purposes…”

## Restrictions to plant biotechnology research in Italy from 2003

Following Legislative Decree No. 224/2003, Law No. 5 was passed on January 19, 2005, to regulate the coexistence between conventional, organic, and GMO agriculture. More specifically, coexistence required that anyone wishing to plant a GMO had to comply with specific ‘Experimental Protocols’ describing the biology, habitat and reproduction of each plant species. Features of the experimental protocols were supposed to be described in Article 3 of the law. However, no deadline was given to draft them and, 19 years later, not a single ‘Experimental Protocol’ has been approved by the Italian Ministry of Agriculture. Without protocols, no GM field trial could be undertaken, not even for the purpose of basic research. Thus, instead of governing coexistence, and despite the fact that the Italian Constitutional Court recognized a principle of coexistence of different crops to guarantee freedom of enterprise and the protection of health and the environment, the law has resulted in a de facto 20-year-long ban by the absence of ‘Experimental Protocols’.

As a result, Italy went from 300 promising field trials during the period between 1992 and 2004 to zero in the following 20 years. Only one field trial with GM cherry, olive, and kiwi trees at the University of Tuscia continued until 2012, but only because it involved automatic renewals of old authorizations. In 2012, Eddo Rugini, the principal investigator for those research projects, was forced to destroy his GM trees, which were just beginning to produce scientific results. As the Italian government decided to ban GMO cultivation for commercial use, it made no sense to sponsor research that might lead to the development of new GMOs. This was a clear message; that there was no future for cultivation of GM plants on Italian soil.

## The farmers’ fight to grow Bt corn

In 2007, while scientific research was still stifled, three farmers from the northeastern region of Friuli—Silvano Dalla Libera, Duilio Campagnolo, and Giorgio Fidenato—began a fight for what they considered their right to plant Bt corn. As farmers were recognized as stakeholders by national regulations, the three Friulians started engaging every level of the national and EU judiciary to defend their rights to plant GM crops authorized in the EU on the basis of these formal interests.

“In 2007, while scientific research was still stifled, three farmers from the northeastern region of Friuli […] began a fight for what they considered their right to plant Bt corn.”

On January 22, 2010, the Italian Council of State, the highest administrative court, sided with Silvano Dalla Libera, recognizing that the state cannot deny, without scientifically solid reasons, the cultivation of Mon810 corn already registered in the EU. His victory marked a decisive turning point: the farmers claimed the possibility to cultivate GMOs, bypassing other potential and more powerful stakeholders such as the hesitant agricultural trade organizations and multinational seed and agrochemical companies.

In spring 2010, Fidenato shared a short video of his field and self-reported to the judicial authorities for cultivating a GM crop without authorization. An inspection by the Forestry Guard verified his claim, and the judiciary began a criminal trial against Fidenato. The judge referred the issue to the CJEU, which recognized the legitimacy of Fidenato’s decision to plant Mon810 corn. Indeed, the cultivation of GMOs such as varieties of Mon810 maize may not be subjected to a national authorization procedure when the use and marketing of such varieties are authorized under Article 20 of EU Regulation No. 1829 and when the same varieties have been included in the common list provided for in Directive No. 2002/53 (establishing the common catalogue of varieties of agricultural plant species), according to the verdict of the CJEU, May 8, 2013.

Meanwhile, on November 16, 2012, as a direct consequence of the trial against Fidenato, a communication from the EU Commission arrived at the Italian Presidency of the Council of Ministers, threatening the country with proceedings due to non-compliance with EU law, namely Decrees No. 212/2001 and No. 224/2003, as well as Law No. 5/2011 of the Friuli Venezia Giulia Region. None of these laws had been notified to the EU, as required by the founding treaty of the European Union. On February 7, 2014 Italy entered into a procedure that might lead to infringement proceedings and, in August 2015, had to remove the criminal penalties from the two laws and reform them to address the criticism from the EU. Thus, the trial against Fidenato indirectly facilitated the legal planting of Mon810 by Silvano Dalla Libera on his farm in 2013 as, since that year, the Italian government was aware that all the three abovementioned Decrees were not in line with European laws.

In the meantime, Fidenato planted Mon810 again in 2014 and self-reported again in opposition to a new Italian Decree, adopted in 2013, preventing Mon810 cultivation on the basis of the emergency measure established in Art. 54 of Reg. 178/2002, the so-called General Food Law Regulation. Fidenato’s field was again destroyed by public authorities, and a new trial began, reaching the CJEU three years later. In the conclusions presented on September 13, 2017, the Court affirmed that this provision “permits the use of emergency measures when it is ‘evident’ that products authorized by that regulation are likely to constitute a ‘serious’ risk to human health, animal health, or the environment.” Thus, Art. 34 does not give member states the possibility to adopt, in accordance with Art. 54 Reg. 178/2002, “emergency measures solely on the basis of” the precautionary principle, “without the conditions set out in Article 34 of Directive No. 1829/2003 being satisfied”. This made the 2013 Italian decree not enforceable.

## The European Union gives more independence to Member States

The question of how to regulate the use of GM crops has always been a contentious issue for the EU. Initially, GMOs were handled by the Directorate-General (DG) for Agriculture, but the responsibility was transferred to DG Health. It later moved to DG Environment, although decisions were often taken by DG Social Affairs. This shift demonstrated that the regulation of GM crops was no longer purely a matter of the life sciences.

In light of the constant arguments, the EU adopted Directive 412/2015, which gives member states the option to ban the cultivation of all types of GMOs on their territory. States can now cite any reason, including issues related to public order, landscape or soil management, but not health or environmental issues, since these are under the purview of the European Food Safety Authority (EFSA) in Parma, Italy. Since the so-called Opt-Out came into effect on April 2, 2015, 19 states have prohibited the cultivation of any type of GMO on their land. On April 22, another communication for amending Regulation (EC) No 1829/2003 proposed that member states can also restrict or prohibit the use of genetically modified food and feed on their territory. The rapporteur expressed “concerns about the far-reaching consequences of the proposal for the functioning of the Internal Market for food and feed and for the competitiveness of the Union’s agricultural sector. As the EU today remains highly dependent on the supply of protein from GM sources and considering that the proposal is likely to have negative indirect effects on imports, Your Rapporteur is of the view that the proposal may seriously endanger livestock production and also negatively affect agriculture in the EU.” For these reasons, the proposal was rejected by the European Parliament, which called on the Commission to withdraw the act: the responsibility for allowing imports of GMOs remains, therefore, a shared EU decision. On April 24, given a positive opinion by EFSA, the Commission authorized the import of 19 additional GM products for both human consumption and animal feed, some of which contain up to eight new introduced genes.

This choice has had two major consequences. While national governments claim to keep their territories GMO-free, Europe imports approximately 83% of the soy it uses as transgenic feed and food. Italy alone imports and consumes five thousand tons of GM soy per day. Thus, most EU countries prohibit GMO cultivation without renouncing the benefits, effectively outsourcing the supposedly environmental and social impacts to other countries. Moreover, some authors (Eriksson et al, 2018; Eriksson et al, 2019) suggested a more coherent and effective Opt-In mechanism, allowing member states to individually authorize the cultivation not only of Mon810 but of all GM plants approved for cultivation by EFSA under Reg. 1829/2003. While ensuring a collective and centralized risk assessment procedure guided by EFSA, this proposal “allows for either adoption or non-adoption of GM crop cultivation in acknowledgment of country-specific arguments that may under certain circumstances favor GM crop cultivation.”

“… most EU countries prohibit GMO cultivation without renouncing the benefits, effectively outsourcing the supposedly environmental and social impacts to other countries.”

## Directive 412/2015 is challenged

Giorgio Fidenato continued to plant Mon810 corn in 2018 and 2021, self-reporting to ultimately reach the European judiciary. In 2018, the field was destroyed by national authorities following a judicial order based on Directive 412/2015 and the subsequent Opt-Out decision adopted by Italy. Fidenato submitted an appeal to the Regional Administrative Court of Friuli Venezia Giulia, which ruled against him. He then appealed to the Council of State, but the court disagreed with him too. His appeal was subsequently discussed by the United Civil Sections of the Supreme Court, which also ruled against Fidenato regarding the compatibility between the cultivation bans on Mon810 and the EU law. Fidenato was thus condemned to pay € 50,000, half of the amount for a field in the province of Pordenone and the other half for a field in the province of Udine. He appealed the fine, and the case was analyzed by Judge Elisa Barro of the Civil Court of Udine, who completely overturned the preceding judgments and considered it necessary to refer the matter back to the CJEU. Judge Barro questioned whether the decision of a member state to deny the authorization for a cultivation that has been authorized at the EU level for decades—without providing any concrete reason—can be considered in line with EU law, and whether this decision does not put Italian farmers at a competitive disadvantage compared to other agricultural entrepreneurs, such as Spanish farmers who have been cultivating Mon810 since 1998. She also questioned whether Regulation 412/2015 conflicts with the free movement of goods, including seeds and products, within the EU.

The Council of State accepted her arguments for referring the case to the CJEU, but the court will undoubtedly face significant challenges in untangling the complexities involved. Meanwhile, the area cultivated with GM crops worldwide increased by 1.9% in 2023 to 206.3 million hectares. 76% of cotton is GM, 72.4% of soybean is GM, 34% of maize, 24% of canola, and 11% of sugar beet are GM plants. In Europe, only Spain cultivates some 50,000 ha with Mon810.

The overall situation is also reflected by the number of experimental field trials in the EU for scientific purposes. Each Member State is asked to notify the European Commission of its intention to conduct a field trial with a GM or NGT plant. The document that must be submitted is the SNIF (Summary Notification Information Format). Once the SNIF is submitted to the EC portal, other Member States can comment within 30 days on the field trial application while the member state in which the application was filed analyzes it. We screened the SNIFs of 14 EU countries that, during the past 22 years, have notified an intention to field-test plants modified by either transgenesis or genome editing (Table 1). Spain leads the EU with 422 out of 940 experimental field requests in accordance with the country’s positive attitude to the use of GM crops. Regarding NGTs, so far only 19 field trials have been notified in the EU by Spain, Belgium, Denmark and Italy, with 10 countries out of 19 that never notified a NGT field trial.

**Table 1:** List of SNIFs submitted to the Member State’s Competent Authorities under Directive 2001/18/EC (after 17 October 2002–until 17 October 2024).

Country	Total	GMOs	NGT	Last 5 y GMOs	Last 5 y	2024 GMOs	2024 NGT
					NGT		
Spain	422	416	6	4	6	0	4
Sweden	79	70	*0*	9	0	2	0
France	77	77	*0*	0	0	0	0
Germany	72	72	*0*	0	0	0	0
Romania	59	59	*0*	0	0	0	0
Czech Republic	33	33	*0*	1	0	0	0
Netherlands	27	27	*0*	0	0	0	0
Belgium	20	14	6	4	6	2	2
Denmark	20	17	*0*	0	3	0	1
Poland	19	19	*0*	0	0	0	0
Portugal	15	15	*0*	0	0	0	0
**Italy**	**10**	**6**	**4**	**0**	**4**	**0**	**4**
Austria	0	0	*0*	0	0	0	0

Countries arranged for total request number. Italy’s peculiar position in highlighted in bold. Overall EU requests:940.

## The legal framework in EU member states

France was the leading agricultural economy in Europe in the 1990s (Kuntz, 2014). GM maize Mon810 cultivation occupied 492.8 ha in France in 2005 and rose to 21,200 ha in 2007. Most of the grain was sold to animal breeders in Spain. Many fields were vandalized, some of them led by the activist Josè Bové, who was later elected as a member of the EU Parliament. The worst incident was the suicide of Claude Lagorce, a farmer at Girac in south-west France, who grew GM maize to feed pigs on his farm in 2007. A flyer announcing an activist demonstration in his field the same day was found next to his body. Finally, the French government banned the cultivation of any GMOs with Law 567 on June 2, 2014.

Two years later, nine French organizations asked to ban herbicide-tolerant rape obtained by mutagenesis: a plant that was exempt from the 2001/18 directive. The dispute with the French government was brought to the CJEU (Gelinsky and Hilbeck, 2018), which ended up dealing with the entire question, including plants obtained through genome editing and ruled that genome-edited plants must be regulated according to Directive 2001/18.

The Netherlands was the first member state to approve a coexistence law with GM crops. Generally, the government and parliament view GM crop varieties as “a very important field of development for the economy and civil society at large.” But the fear of protests from environmentalist groups blocked field trials. In 2008, a local Commission on Genetic Modification said that “given the small area of land used for agriculture in the Netherlands, the GM crops currently available are not particularly attractive for Dutch farming.” While no GM crop is cultivated in the Netherlands, the country is importing GM products and redistributes them to other EU and non-EU countries.

Spain is currently the only EU member state in which GMOs are grown after it first authorized cultivation of GM maize in 1998. Since, 1.65 million hectares have been planted with Mon810 Bt-corn with economic and environmental benefits for farmers (Bertho et al, 2020; Brookes, 2019). Lately, the area cultivated with Bt-corn has decreased, partially in line with the overall decrease of maize cultivation in Spain, and in part as no new varieties are authorized in the EU. Spain’s relatively benevolent tradition on GMOs is still valid though with the country leading in the number of field trials of new plant genotypes and SNIF notifications for both GMOs and NGTs (Table 1).

“Spain’s relatively benevolent tradition on GMOs is still valid though with the country leading in the number of field trials of new plant genotypes and SNIF notifications for both GMOs and NGTs.”

In Germany, field trials with GMOs are subject to the strict *Gentechnikgesetz* (genetic engineering law). From 1994 to 2007, more than 300 field trials with 15 different GM oilseed rape events took place at 88 different sites in Germany (Belter, 2016). However, Germany became increasingly restrictive to the use of plant biotechnology and there have been no field trials requests since 2013.

We finally want to mention Poland where no GMOs are cultivated and no application for field trials have been filed, but where new restrictive measures have been passed to reduce import of GM feed, to protect national maize production. The ruling, that was supposed to start taking place in 2025, has been postponed to 2030, possibly for concerns about supplying an adequate amount of good quality non-GM feed.

## The CRISPR paradox

With the development of genome-editing technologies based on CRISPR/Cas, several plant scientists, who had worked for years under the ban on GMO cultivation, reignited the debate about plant genetic modification. A key feature of this system is the ability to introduce small, targeted changes in a genome without permanently integrating a transgene to confer the desired characteristics. CRISPR challenged existing EU laws because plants obtained by editing specific nucleotides are indistinguishable from those obtained by spontaneous mutations that are not regulated. In 2016, the authors Fabio Fornara and Vittoria Brambilla asked the Italian Ministry of the Environment whether plants obtained by editing were subject to the same regulations applied to classical GMOs and, if not, which procedures to follow for field experiments. National authorities were in a legal vacuum, waiting for the EU.

A partial answer on the equivalence between edited plants and GMOs was provided by the CJEU in July 2018. The Court affirmed that gene editing gives rise to a GMO, according to the legal definition in Article No. 2 of Directive 2001/18/EC, and therefore, edited plants fall under GMO regulations. This interpretation was not surprising: the CJEU provides assessments based on existing laws but cannot extend beyond the meaning of the law to create a new one. In addition, genome editing could not be exempted from the Directive due to its novelty. On the contrary, the CJEU exempted older genetic manipulation techniques, such as random mutagenesis, that have been in use for commercial production half a century before Directive 2001/18, describing them as having a “long safety tradition.” This ruling created a curious legal scenario in which the same genotypic trait could be introduced by conventional methods or by gene editing, modifying the same DNA nucleotides. However, while the former could be registered and commercialized following an easier path, the latter would require a very expensive and complex procedure, effectively discouraging the cultivation of genome-edited plants. This major contradiction results from the basic notion of the Directive, which regulates the methods rather than the products obtained with them. We argue that such an approach is not sustainable in the long term as technological progress undermines the basic tenets of the law.

## A new opportunity for scientists

Since the 2018 CJEU judgment that classified gene-edited plants as GMOs, Italian scientists have continued to produce CRISPR mutants in various plant species and have started an active dialogue with politicians and society. In an effort to improve communication and dissociate from the term GMO, the Italian Society of Agricultural Genetics (SIGA) proposed a different and ‘easier’ name for the new genomic techniques: ‘Assisted Evolution Techniques’ (TEA). TEA emphasizes technologies rather than the organism itself, making the term resilient to technological advancements, unlike the legal definition of GMO. Among the few Italian organizations that supported these issues, a significant contribution came from the Luca Coscioni Association for Freedom of Scientific Research.

**Figure 1:**
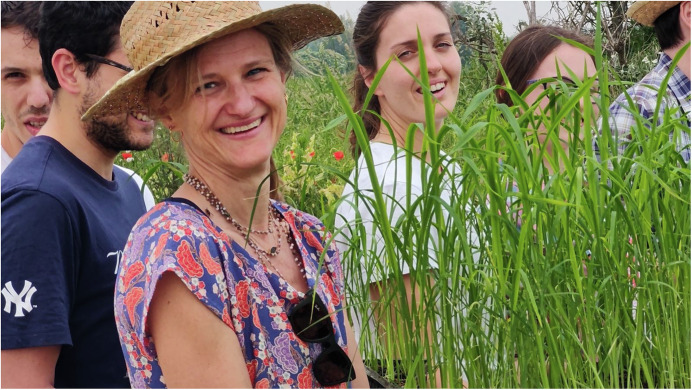
Federico Mirone, Giulio Vicentini, Vittoria Brambilla, Greta Bertagnon, Giulia Ave Bono and Lorenzo Mineri (from left to right) planting the first gene-edited rice plants from the Ris8imo project in an open field trial on May 13, 2024 in Mezzana Bigli, Italy.

In 2023, following the European Commission’s July 2023 document on NGTs, and with the support from all three major Italian agricultural trade organizations—Confagricoltura, Coldiretti and CIA—the Italian Parliament decided to support gene editing in plant breeding. The decision was made to facilitate applications for field trials of plants obtained through genome editing. Interestingly, notifications for open trials of NGT plants to the Ministry of the Environment did not require passing a new law but involved a simple ‘editing’ of the Legislative Decree No. 224/2003 by removing just the 176 characters that referred to the Experimental Protocols. The removal of this legislative barrier allowed Vittoria Brambilla to request permission to test a rice variety that is less susceptible to a fungal pathogen causing rice blast disease. The experiment, called RIS8imo, suggesting a good Italian risotto, started with rice plantlets being transplanted in the field on the 13th of May 2024 (Fig. 1) and was supported by Fondazione Bussolera Branca and led by Fabio Cei and Roberto Schmid and adhered to the health and environmental impact dictated by a law originally conceived against GMOs. After Ris8imo, a second NGT-1 field trial named ViTEA was authorized in Italy in September 2024: downy-mildew-resistant grapevines were transplanted in the wine-growing area of Valpolicella by a research group lead by Sara Zenoni and Mario Pezzotti from the University of Verona and owners of the spin-off company EdiVite (Meldolesi, 2024).

“In 2023, following the European Commission’s July 2023 document on NGTs, and with the support from all three major Italian agricultural trade organizations […] the Italian Parliament decided to support gene editing in plant breeding.”

“The removal of this legislative barrier allowedVittoria Brambilla to request permission to test a rice variety that is less susceptible to a fungal pathogen causing rice blast disease.”

The legal and social debate over the use of genetic technologies in agriculture has reached a tipping point. Local and national administrators, agricultural trade organizations, farmers, and scientists have united in a common effort to use technological innovations in agriculture to serve farmers and consumers. At the European level, NGTs are proposed to be distinguished into two categories. NGT-1 are plants indistinguishable from those produced through conventional breeding methods and therefore subject to simplified approval procedures whereas NGT-2 would fall under the regulatory regime for ‘classic’ GMOs.

“The legal and social debate over the use of genetic technologies in agriculture has reached a tipping point.”

The impact on Italian plant science is exemplified by the EU registry of GMO field trials, with four out of the last eight requests coming from Italy, all based on NGT. The RIS8imo rice experiment, was conducted on the farm of Federico Radice Fossati who has expressed his availability in hosting further field trials, also from other EU countries, received unexpected interest and support from citizens, as well as coverage in the news. This interest exponentially increased after the devastation of the experimental field by unknown vandals on the night of the summer solstice in 2024, which made the news on prime-time television. It shows that the dialogue with society still has problems, but also that times have changed: while the destruction had no repercussions, solidarity and support for scientists came in abundance from different parts of society. The vandals though didn’t kill all rice plants; the survivors recovered and have completed the vegetative cycle, being able to produce seeds that will soon be analyzed.

## Conclusions

For democracy to work, citizens need to be informed and consulted, as highlighted by the experience of the European Green Papers, which promote the active involvement of citizens in decision-making processes. The complex situation regarding the use of GM crops in agriculture illustrates the challenges our policymakers, legislators, courts, but also food business, scientists, and the entire civil society, are asked to face in the future. The balance between protection of the environment, consumers’ health, and economic interests cannot necessarily and ideologically be put in contrast with innovation and scientific progress, as it happened for GMOs. On the contrary, regulatory and policy decisions should consider all the interests and rights at stake and should be able to effectively and correctly communicate the political, economic, scientific, and ethical issues. This can be ensured, first and foremost, through dialogue between law and science to guarantee that regulatory choices properly take into consideration scientific knowledge and to regulate progress—and scientific research—in an effective and clear way.

“The balance between protection of the environment, consumers’ health and economic interests cannot necessarily and ideologically be put in contrast with innovation and scientific progress, as it happened for GMOs.”

Balancing the different rights and interests in a complex context such as agri-food, characterized by strong resistance, skepticism, but also a strong need to boost innovative solutions to address urgent issues such as climate change and sustainability, is a difficult challenge. But the legislators are, now more than ever, called upon to seriously address regulatory solutions. The heated debate between different governance levels—EU and member states—as well as between the scientific community and policymakers has created a confused situation, involving different institutional actors—legislators but also courts at different levels—that often resulted in tensions and controversial approaches. The consequent contradictions and uncertainties that have emerged from a “never ending” judicial and legislative querelle on gene-editing techniques and GMOs have created—and still create—confusion and disorientation in business operators—farmers *in primis*—as well as in civil society and consumers.

This situation negatively affects, as clearly underlined in these pages, scientific progress, consumers’ rights and freedom of economic initiative, which are damaged by the lack of a comprehensive, stable and clear regulatory approach and institutional response. Facing the challenges concerning NGTs and GMOs through the promotion of a serious, informed and open-minded dialogue amongst diverse actors and stakeholders seems to be the only solution to ultimately ensure active and informed involvement of citizens in the decision-making process, thus protecting the key values on which our democracy is founded.

## Supplementary information

Peer Review File
