# Addendum: The history of GM crops in Italy

**DOI:** 10.1038/s44319-024-00359-4

**Published:** 2025-01-08

**Authors:** Roberto Defez, Maria Chiara Errigo, Giulia Formici, Lucia Scaffardi, Eleonora Sirsi, Fabio Fornara, Vittoria Brambilla

**Affiliations:** 1 Institute of Biosciences and Bioresources (IBBR), CNR, Napoli, Italy; 2 Department of Law, Politics and International Studies, University of Parma, Parma, Italy; 3 Department of Law, University of Pisa, Pisa, Italy; 4 Department of Biosciences, University of Milan, Milan, Italy; 5 Department of Agricultural and Environmental Sciences - Production, Landscape, Agroenergy, University of Milan, Milan, Italy

**Keywords:** Economics, Law & Politics, History & Philosophy of Science, Plant Biology

## Abstract

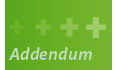

**Addendum to:** *EMBO Reports* (2024) 26(1):9–15. 10.1038/s44319-024-00330-3 | Published online 8 January 2025

Our manuscript (10.1038/s44319-024-00330-3) focuses on legislative steps and political decisions that marked key moments in the history of agricultural biotechnologies in Italy. However, we believe it is also important to highlight the role and contributions of scientists who made these developments possible.

In 2000, the Italian Society of Agricultural Genetics (SIGA) entered the public debate on GM technologies by publishing a document aimed at addressing misconceptions and prejudice, while highlighting the benefits, safety, and potential of GM crops (http://www.geneticagraria.it/attachment/SocietaScuolaRicerca/AlimBiotec.pdf). This document targeted broader society and fostered a debate that has been continuing ever since. In retrospect, it is noteworthy how SIGA addressed arguments against agricultural biotechnologies that have remained unchanged for 25 years, in sharp contrast with the rapid advancement of several technologies.

In 2004, the Italian Toxicology Society (SITOX) coordinated a consensus document among 15 academies and scientific societies regarding the safety of GM food (https://archivio2.sitox.org/docs/Consensus.pdf ). The proponents included almost all Italian scientists working in the life sciences. The document analyzed available studies on the potential risks associated with the use of GM food and feed and found no specific concerns. It also laid out how the regulatory framework guaranteed more rigorous safety tests compared to conventional crops. The document emphasized several aspects we believe to be crucial in both regulatory assessments as well as social debates, including the need to view science as continuously evolving rather than static, to regard science as a service to humanity, and to recognize that no technology is perfect on its own, but its success or failure depends on a careful evaluation of its risks and benefits.

In 2006, SITOX coordinated a second consensus document involving 21 academies and scientific societies, revising the criteria for coexistence among GM, conventional and organic crops (https://archivio2.sitox.org/docs/sitox_comunicato_ogm_2006.pdf). This document was primarily based on studies conducted on maize and concluded that coexistence among different agricultural practices is feasible, with the levels of admixture between products well below the limits established by the EU. In the context of the potential adoption of gene-edited crops, these insights gained from experience with transgenic crops could help shape future regulations.

Following the first successful application of the CRISPR/Cas9 system to several plant species in 2013, scientists continued to engage in dialogue with society and policymakers, which reignited the social debate over genetic modification in agriculture. Recognizing the importance of social acceptance and building on the experience gained from the GMO debate, SIGA initiated several communication efforts. Many of these took place in Milan during the 2015 EXPO, where the main theme, “Feeding the Planet, Energy for Life,” placed agriculture at the center of discussions about future challenges and called upon plant scientists to propose solutions. As part of its commitment to society and outreach, SIGA established a permanent communication group in 2015 to provide a scientific perspective on issues related to agriculture, food, and biotechnologies.

With the exponential growth of studies related to genome-editing applications in plants, expectations related to this new technology increased. In 2017, SIGA published *Prima i Geni* (Genes First), a manifesto in support of genome editing (http://www.geneticagraria.it/attachment/SocietaScuolaRicerca/Manifesto_Prima_i_Geni.pdf). It is framed around several key messages and tailored to an audience without scientific training. The document highlights time as a crucial factor in shaping the social perception of technological innovations in agriculture: it transforms technological advancements into traditions in a continuous and ongoing process. Thus, biotechnological innovations are not an end goal but rather one of many tools available for enhancing well-being. Genome editing is part of this process, and its value lies in accelerating what agriculture has been pursuing for millennia. The document also proposed an Italian pathway for utilizing genetic innovations, leveraging the country’s rich biological and food diversity. To promote the technology among a specialized audience, SIGA organized hands-on courses for scientists, with the first edition held in Turin in 2018, and repeated yearly in other cities. The course has so far attracted dozens of young scientists interested in applying editing technologies.

The collaborative efforts between scientists, society, and agricultural organizations led to the signing of the *Camici e Trattori* (Lab Coats and Tractors) agreement by SIGA and Coldiretti in Rome on June 17, 2020. Coldiretti, an influential farmers’ association, joined forces with SIGA to promote editing technologies and a scientific approach to meet contemporary challenges in agriculture. The agreement has positively impacted on the social and political dialogue, facilitating subsequent political decisions.

