# Supplementary material for: The history of GM crops in Italy: After two decades of a de facto ban, the engagement of farmers and scientists has prompted the Italian government to allow field-testing of NGT/TEA plants again
Source: EMBO Rep. 2024 Nov 27;26(1):9–15. doi: 10.1038/s44319-024-00330-3 (PMC11724101; doi:10.1038/s44319-024-00330-3)
Supplement: Supplementary file 1 — Peer Review File [file 44319_2024_330_MOESM1_ESM.pdf]

EMBO reports

The history of GM crops in Italy

Roberto Defez, Maria Chiara Errigo, Giulia Formici, Lucia Siffardi, eleonora sirsi, Fabio Fornara, and Vittoria Brambilla

Corresponding author(s): Vittoria Brambilla (vittoria.brambilla@unimi.it) , Roberto Defez (roberto.defez@gmail.com)

Review Timeline:

Submission Date:	28th Sep 24
Editorial Decision:	8th Oct 24
Revision Received:	27th Oct 24
Accepted:	15th Nov 24

Editor: Holger Breithaupt

Transaction Report: This article was editorially reviewed.
